# An Easy-to-Use Approach to Detect CNV From Targeted NGS Data: Identification of a Novel Pathogenic Variant in MO Disease

**DOI:** 10.3389/fendo.2022.874126

**Published:** 2022-06-28

**Authors:** Serena Corsini, Elena Pedrini, Claudio Patavino, Maria Gnoli, Marcella Lanza, Luca Sangiorgi

**Affiliations:** Department of Rare Skeletal Disorders, Istituto di Ricovero e Cura a Carattere Scientifico (IRCCS) Istituto Ortopedico Rizzoli, Bologna, Italy

**Keywords:** CNV detection, Targeted NGS data, Rare skeletal disease, Multiple Osteochondromas, EXT1, EXT2

## Abstract

**Background:**

Despite the new next-generation sequencing (NGS) molecular approaches implemented the genetic testing in clinical diagnosis, copy number variation (CNV) detection from NGS data remains difficult mainly in the absence of bioinformatics personnel (not always available among laboratory resources) and when using very small gene panels that do not meet commercial software criteria. Furthermore, not all large deletions/duplications can be detected with the Multiplex Ligation-dependent Probe Amplification (MLPA) technique due to both the limitations of the methodology and no kits available for the most of genes.

**Aim:**

We propose our experience regarding the identification of a novel large deletion in the context of a rare skeletal disease, multiple osteochondromas (MO), using and validating a user-friendly approach based on NGS coverage data, which does not require any dedicated software or specialized personnel.

**Methods:**

The pipeline uses a simple algorithm comparing the normalized coverage of each amplicon with the mean normalized coverage of the same amplicon in a group of “wild-type” samples representing the baseline. It has been validated on 11 samples, previously analyzed by MLPA, and then applied on 20 patients with MO but negative for the presence of pathogenic variants in *EXT1* or *EXT2* genes. Sensitivity, specificity, and accuracy were evaluated.

**Results:**

All the 11 known CNVs (exon and multi-exon deletions) have been detected with a sensitivity of 97.5%. A novel *EXT2* partial exonic deletion c. (744-122)-?_804+?del —out of the MLPA target regions— has been identified. The variant was confirmed by real-time quantitative Polymerase Chain Reaction (qPCR).

**Conclusion:**

In addition to enhancing the variant detection rate in MO molecular diagnosis, this easy-to-use approach for CNV detection can be easily extended to many other diagnostic fields—especially in resource-limited settings or very small gene panels. Notably, it also allows partial-exon deletion detection.

## Introduction

The advent of new technologies in recent years, particularly in the field of next-generation sequencing (NGS), has improved and increased the possibility of identifying single-nucleotide variants and small deletions/insertions responsible for numerous pathologies (1), including hereditary skeletal diseases. At the same time, the role of copy number variations (CNVs) as a causative genetic factor for many disorders (2) makes their detection essential for complete diagnostic approaches.

Currently, the gold standard for CNV detection is the Multiplex Ligation-dependent Probe Amplification (MLPA), which has, however, some limitations related both to the technique (i.e., CNVs in genetic regions not covered by the probes remain undetected) and to a non-exhaustive availability of kits, which leaves some genes unanalyzable. To overcome these limitations, many NGS-related tools have been developed (3, 4) to identify CNV variants, thus making molecular screening more complete and cost- and time-saving. Various software have been developed for this purpose, although having some critical issues; most of them are oriented toward wide genomic regions analyses (i.e., whole-genome, whole-exome, and large NGS panels), showing a lower performance on data deriving from smaller NGS panels, which, in turn, are the most used and allow more precise identification of CNVs at a single or few exons level (4). In addition, they require additional costs and/or specialized bioinformatics personnel, not always available within laboratory resources. It must also be considered that, in the case of very small NGS panels, the design cannot be optimized for CNV detection.

In this study, we propose an “easy-to-use approach” to detect heterozygous single- and multi-exon CNVs from NGS data, not requiring specialized bioinformatic skills or the purchase of specific software. We reported our experience in the multiple osteochondromas (MO) disease whose diagnosis is based on the use of an Ion Torrent S5 NGS assay using a very small (two-gene, 12.78-kb) gene panel. Although being a rare genetic disorder with an estimated prevalence of 1:50,000 (5), MO is among the most common inherited musculoskeletal diseases (6). It is a dominant autosomal hereditary condition caused by heterozygous mutations in exostosin-1 (*EXT1*) or exostosin-2 (*EXT2*); *EXT* causative variants have been reported in 70%–94% of patients with MO (7). The majority (80%) of mutations are nonsense, frameshift, or splice-site mutations, resulting in the truncation of the EXT1 or EXT2 protein (8); other detected causative variants are missense mutations, resulting in loss of EXT activity (9), and large deletions —exon or multi-exons— accounting for up to 8% of the cases (10–13). According to the *EXT1/EXT2* mutation database (https://databases.lovd.nl/shared/genes/EXT1, https://databases.lovd.nl/shared/genes/EXT2), *EXT1* is characterized by a prevalence of multi-exon deletions (37%), whereas *EXT2* has most single-exon deletions (45%) (**Supplementary Figure 1**). Despite this, 10%–15% of patients remain undiagnosed, suggesting the possible presence of other MO-causative genes or not detected *EXT* variants.

Here, we describe an “easy-to-use” algorithm validated and optimized to detect CNVs using NGS data from a very small (12.78-kb) targeted gene panel. The pipeline permitted to identify a novel MO-related large deletion involving a portion of intron 4 and exon 5 of *EXT2*, not previously detected by MLPA technique, thus providing a new diagnostic tool to improve MO diagnosis.

## Materials and Methods

### Patients

This project was approved by the CE-AVEC (Comitato Etico di Area Vasta Emilia Centro della Regione Emilia-Romagna) ethics committee (20-12-2017/No. 0012819). All research was performed in accordance with the relevant guidelines/regulations. A total of 155 patients with MO were enrolled in the study. All patients were clinically and genetically diagnosed at the Center of Rare Skeletal Disease of the Istituto Ortopedico Rizzoli (IOR, Bologna, Italy). DNA was extracted from peripheral blood through an automated NX^P^ Biomek platform (Beckman Coulter Life Science). All patients underwent molecular evaluation for the presence of pathogenic variants in the *EXT1* and *EXT2* genes following the diagnostic protocol described in our previous studies (11, 14) using Denaturing High-Performance Liquid Chromatography (DHPLC) and Sanger sequencing to detect the point mutations, and MLPA to detect big deletions/insertions. Considering all 155 patients, 11 (Samples 1–11) were characterized by the presence of large deletions in the *EXT* genes detected by MLPA, 124 (Samples 12-135) carried a pathogenic *EXT* point mutation detected by DHPLC, and 20 (Samples 136–155) were negative on *EXT* molecular screening **Table 1**. As detailed in **Table 2**, the 11 CNV-positive control samples are characterized by single-exon or multi-exon deletions in *EXT1* or *EXT2*. As control samples to evaluate the pathogenicity of the new variant identified in the study, we also enrolled two relatives of Sample 146 (Relative 1 and Relative 2), both clinically affected by MO.

**Table 1:** Dataset description: genetic and NGS qualitative metrics results.

Samples	Count
**Total**	**155**
**Genetic results by DHPLC/MLPA molecular screening**	
*EXT* Point variants (by DHPLC/Sanger sequencing)	124
*EXT* CNV variants (by MLPA)	11
No *EXT* variants	20
**NGS data quality**	
“Mean depth” > 100× and uniformity > 90%	152
“Mean depth” < 100×	2
Uniformity < 90%	1

**Table 2:** Detail of the CNVs described in the study on the basis of the MLPA analysis and the algorithm developed.

ID	Gene	CNV Description	Genome Ref. (MLPA Results)	Genome Ref. (CNV Algorithm)
Sample 1	*EXT1*	Del exon 1	g.119123222-119123291	g.119122772-119123839
Sample 2	*EXT1*	Del exon 1	g.119123222-119123291	g.119122772-119124113
Sample 3	*EXT1*	Del exon 1	g.119123222-119123291	g.119122772-119124113
Sample 4	*EXT1*	Del exon 1	g.119122566-119123291	g.119122108-119124113
Sample 5	*EXT1*	Del exons 2–4	g.118842473-118849325	g.118842223-118849515
Sample 6	*EXT2*	Del exon 8	g.44193218-44193291	g.44192994-44193383
Sample 7	*EXT1*	Del exons 1–8	g.118825129-119123291	g.118825037-119124113
Sample 8	*EXT2*	Del exon 8	g.44193218-44193291	g.44192994-44193383
Sample 9	*EXT1*	Del exons 2–11	g.118811842-118849325	g.118811558-118849515
Sample 10	*EXT2*	Del exon 8	g.44193218-44193291	g.44192994-44193383
Sample 11	*EXT2*	Del exon 3	g.44130744-44130814	g.44130570-44130960
Sample 146	*EXT2*	Partial del exon 5	Not detected	g.44146239-44146376

### NGS Analysis

All 155 patients with MO were re-evaluated by NGS using the Ion Torrent PGM platform (Thermo Fisher Scientific, Waltham, MA, USA). An *EXT1-EXT2* custom panel was designed using the Ion AmpliSeq Custom Designer (https://www.ampliseq.com). The panel is composed of two primer pools covering all UTRs (Untranslated regions) and coding regions of *EXT1* and *EXT2* genes (12.78 kb), also including at least 50-bp flanking intronic regions. The amplicon range is 125–275 bp, and the expected coverage is 99.37%. All amplicons and their target regions are detailed in **Supplementary Table 1**.

Each sample was processed using the Ion AmpliSeq Library Kit 2.0 (Thermo Fisher Scientific); the Ion Chef platform (Thermo Fisher Scientific) was used to automate the template preparation and to load the Ion 314/316 Chip Kit v2 BC. The sequencing reaction was then performed on the Ion Torrent PGM platform. NGS data were analyzed using the Torrent Suite Software (Thermo Fisher Scientific) and the SEQNext application (JSI medical systems GmbH, Germany).

### Baseline Creation

The group of 124 samples carrying pathogenic *EXT* point mutations was used to create the baseline needed to define the mean sequencing depth (normalized to the total number of reads) of each amplicon in the “wild-type” (i.e., CNV-free) samples. To be included in the baseline, samples were selected on the basis of NGS data quality, considering mean depth and uniformity. All samples with a mean coverage of <100× or uniformity of <90% were excluded to avoid uneven coverage caused by a poor efficiency of some primers **Table 1**.

To evaluate the most reliable number of samples to build the baseline, we created and tested four baselines starting from a different sample size:

- 10-sample baseline
- 30-sample baseline
- 50-sample baseline
- 100-sample baseline

This evaluation is necessary to assess whether the pipeline robustness, which depends on the baseline, is related to the use, and therefore the availability, of a large number of wild-type (non-CNV–carrying) samples. In this case, the CNV pipeline could be hardly usable in case of a limited patient flow (e.g., ultra-rare diseases and non-reference center for rare diseases). In addition, to estimate whether sample selection affects the quality of the baseline (depending on samples’ “coverage” or “uniformity” parameters), for each sample size (10, 30, 50, and 100), we tested the baseline using five different iterations randomly generated; each iteration will consist of a different selection of samples. Overall, 20 different baselines (five iterations for four different sample sizes) were created. Each baseline was then tested on the positive control group to define its robustness; the most reliable will be used to analyse the 20 “undiagnosed” MO samples.

### CNV Detection Algorithm

The algorithm used to detect CNVs using NGS data is based on a simple comparison between the amplicon depth (normalized to the total number of reads) of the investigated sample and the mean depth (normalized) of the same amplicon defined in the baseline.

For each amplicon, the ratio “depth (normalized)/average baseline depth (normalized)” was calculated and reported as a percentage value. As defined by previous studies (15, 16), a constitutional heterozygous deletion is defined by a value of <75%. Different thresholds (i.e., 70%, 65%, and 60%) were tested to increase specificity and assess the detection limit of the algorithm.

### Validation of the CNV Tool

To evaluate the performance of our CNV pipeline, we calculated the sensitivity, specificity, and accuracy, considering the 11 known CNV-positive samples (Samples 1–11) described in **Table 2**. For each NGS amplicon that overlaps the MLPA target regions (detailed in **Supplementary Table 2**), we calculated the ratio “depth (normalized)/average baseline depth (normalized)”, considering all 20 baselines; we assigned each amplicon a “correct” or “wrong” value, considering the concordance with the MLPA results. A “true positive” (TP) or “true negative” (TN) value were assigned in case of a match with the MLPA analysis. “False positive” (FP) value was attributed when the CNV tool called a variant not detected by MLPA, whereas a “false negative” (FN) value if the tool did not detect a CNV revealed by MLPA. Sensitivity was calculated as TP/(TP + FN), specificity as TN/(TN + FP) and accuracy as (TP + TN)/(TP + TN + FP + FN). To compare the different baselines, sensitivity, specificity, and accuracy were calculated for each iteration. Sensitivity, specificity, and accuracy were also calculated considering a threshold different from <75% for the “deletion” call (i.e., <70%, <65%, and <60%); these latter values are reported only as the average of the five iterations in the Results section.

### CNV Analysis

The 20 samples (Samples 136–155) negative to the presence of *EXT* variants using the DHPLC-MLPA approach were reanalyzed by NGS and then evaluated for the presence of CNVs using the validated pipeline. Real-time qPCR (quantitative Polymerase Chain Reaction) was performed to validate all “positive” calls involving amplicons outside the regions covered by the MLPA probes, as well as in the event of a discordant result with MLPA. The reaction was realized using RT2 SYBR^®^ Green qPCR Mastermix (Qiagen, GmbH, Hilden, Germany) and a pair of primers specifically designed on the region identified as deleted. Primer sequences and thermocycler conditions are available under request. The pathogenicity of the variant was investigated by evaluating its presence in two affected relatives.

## Results

### NGS Analysis

All samples selected for the study were analyzed using the *EXT1/EXT2* targeted NGS panel. For each sample, the mean depth, the uniformity of coverage, the on-target percentage, and the number of mapped reads are detailed in **Supplementary Table 3**. All pathogenic mutations previously detected have been confirmed, as well as the absence of MO-causative variants (i.e., point mutations and small indels) in the 20 patients with genetically “undiagnosed” MO (Samples 136–155).

Applying the selection criteria (i.e., “mean coverage” of >100× and “uniformity” of >90%), 121 out of 124 CNV-free samples (Samples 12–135) were eligible to create the baseline. Sample 31 was discarded since having a uniformity value of 54.15%, whereas Sample 90 and Sample 128 were excluded due to the low coverage (37.48× and 8.39×, respectively); the remaining samples were characterized by a mean depth of 620× (range: 175.8–1,538×) and a mean uniformity of 92.89 (range: 91.1%–95.6%). Through random selection, 20 groups of samples (five different selections of 10, 30, 50, and 100 sample size) were generated (detailed in **Supplementary Table 4**) and then used to create 20 different baselines.

### Validation of the CNV Pipeline

Method validation was performed with 11 CNV-positive control samples. Considering only the amplicons overlapping the MLPA target regions, sensitivity, specificity, and accuracy were calculated on a total of 429 individual genetic regions (11 samples, 39 amplicons per sample) (data derived from **Supplementary Table 2**). Two amplicons (AMPL7153865992 and AMPL7156300405) were characterized by a systematic error; notably, they are in the *EXT1-UTR* region, outside the relevant diagnostic area.

The positive control samples were evaluated using all the 20 created baselines to evaluate the most effective ones. All results are detailed in **Supplementary Table 5** as follows: all 'depth (normalized)/average depth (normalized)' ratios of <75% are reported, whereas values of >75% are reported only in case of discrepancy between MLPA and NGS-based CNV pipeline.

**Figure 1:**
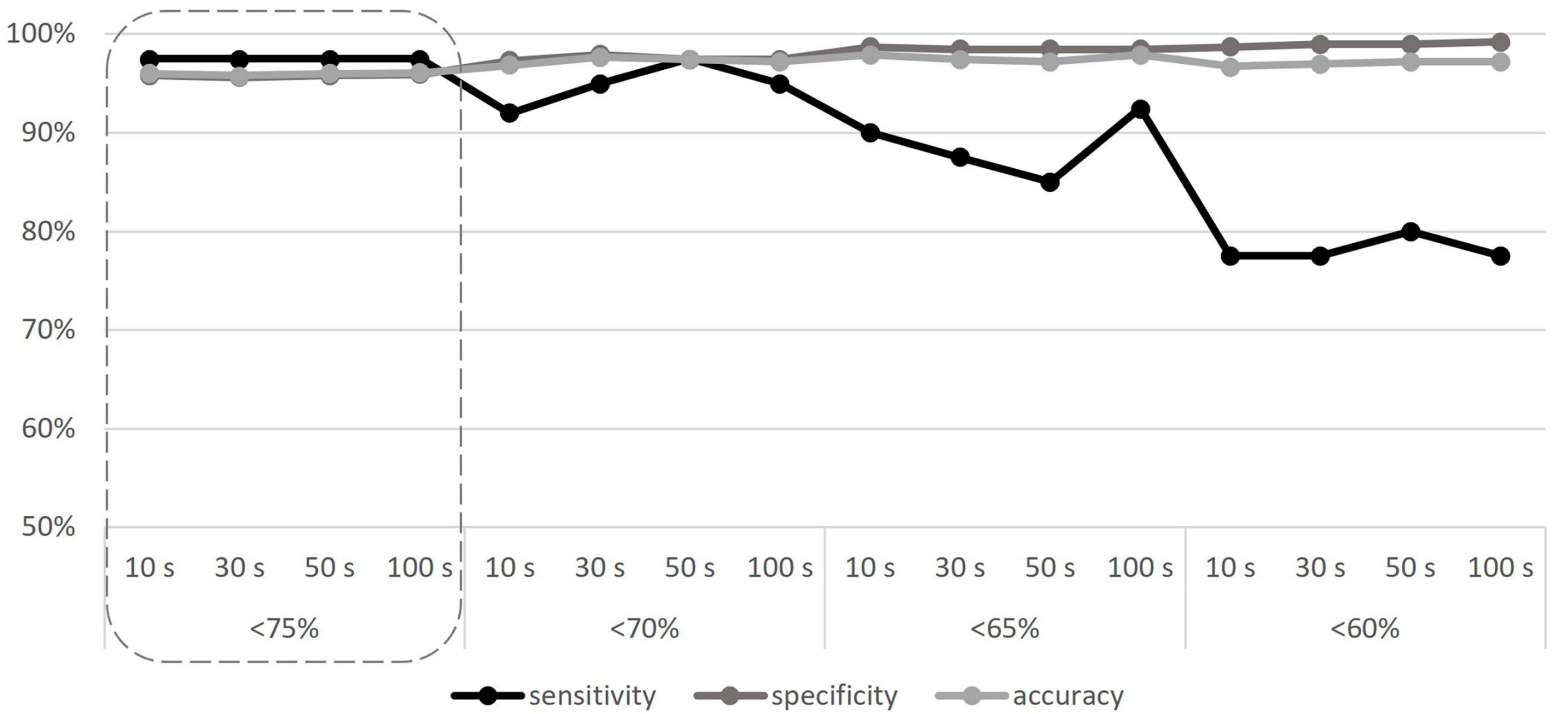
Sensitivity, specificity, and accuracy calculated according to different baseline sample sizes (10, 30, 50, and 100 samples), and different CNV detection thresholds (<75%, <70%, <65%, and <60%). The adequate threshold value is highlighted by the dashed line. S, samples.

With all the baselines, the CNV pipeline detected the 11 known CNVs even if AMPL7153088419 always failed to detect the deletion in Sample 9, attesting the sensitivity of the algorithm to 97.5% (as detailed in **Supplementary Table 6**); despite this, all other 14 amplicons that cover the MLPA target regions (known to be deleted in Sample 9) confirm the results (see **Supplementary Table 5**), thus making the only detected false positive not relevant to the final result. Considering the specificity, we detected a number of false positives ranging from 14 to 20, depending on the baseline; the numbers of “false positives”, “false negatives”, “true negatives”, and “true positives” related to each baseline are detailed in **Supplementary Table 6**, providing a mean specificity and average total accuracy, respectively, of 95.84% (95.37%–96.40%) and 95.99% (95.57%–96.50%) using the 10-sample baseline, 95.63% (94.86%–96.40%) and 95.80% (95.10%–96.50%) using the 30-sample baseline, 95.84% (95.12%–96.14%) and 95.99% (95.34%–96.27%) using the 50-sample baseline, and 95.94% (95.37%–96.14%) and 96.08% (95.57%–96.27%) using the 100-sample baseline. According to our results, we did not find any significant differences using baselines built on different sample sizes, as well as considering different samples selections, thus making the use of 10 samples—randomly selected—sufficient. To increase specificity, thus reducing the number of “false positive” calls that require confirmation in case of negative results, we repeated the analyses considering three different thresholds for the “deletion” call (<70%, <65%, and <60%). Results obtained are represented in **Figure 1** and described in **Supplementary Table 7**, considering, for each baseline, the average values obtained by the five different iterations. Although the increase of specificity as the threshold decreases is expected (up to 99.23% for the 60% threshold), a reduction of sensitivity is evident, as follows: 92%–97.50% (depending on the baseline) for the <70% threshold, 85%–92.50% in the case of the <65% threshold, and 77.50%–80% for the <60% threshold. Despite the choice to consider the “deletion” threshold at <75%, it should be emphasized that even with the “lower sensitivity” threshold (<60%), all 11 deletions continue to be detected, although not in their entire length.

By evaluating all the results, and not just those corresponding to the MLPA target regions, the deletions described by this tool comprise an actually larger gene region, as detailed in **Table 2** and shown in **Supplementary Table 5**. Considering the 11 samples, the smallest deletions detected by the CNV pipeline are covered by two contiguous amplicons: (AMPL7156321442 + AMPL7156321443) for Sample 11 and (AMPL7155115758 + AMPL7155115759) for Samples 6, 8, and 10.

### CNV Analysis

**Figure 2:**
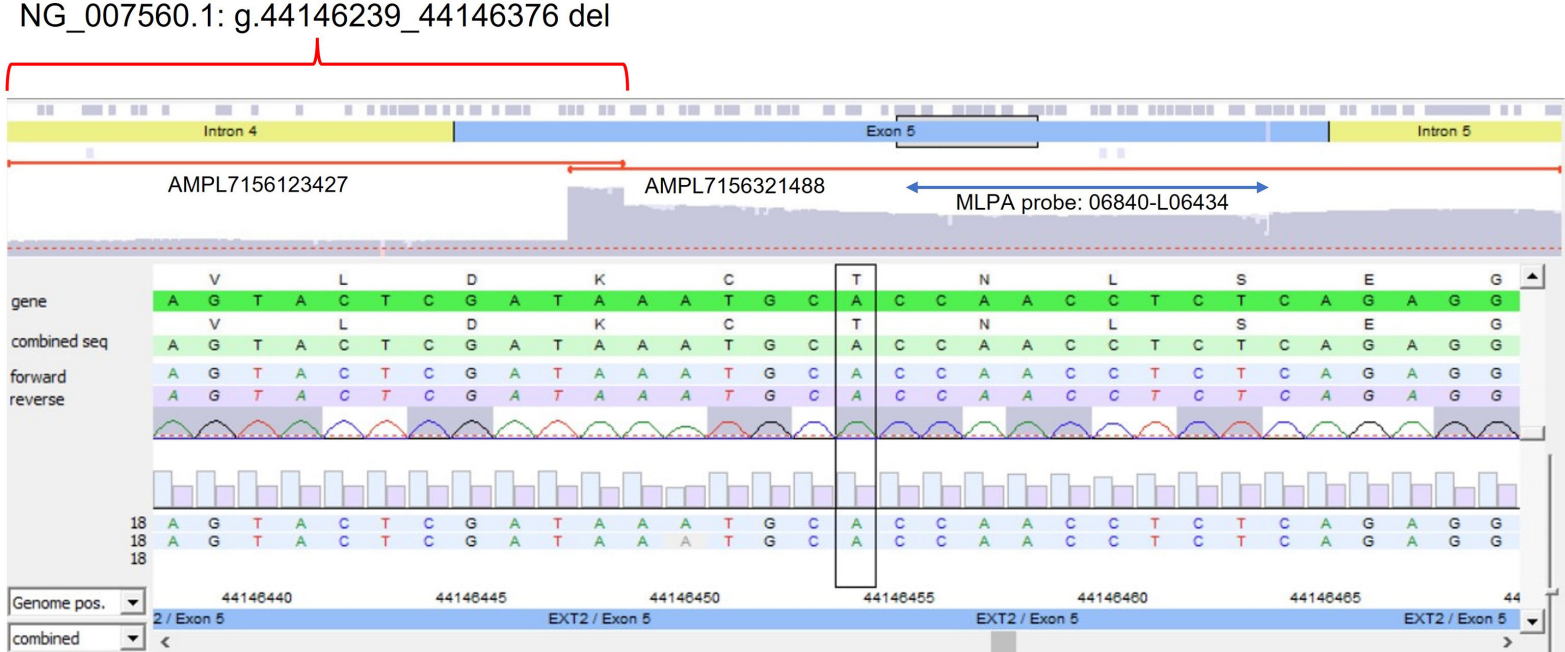
Novel partial exon 5 deletion detected in *EXT2* gene. The deleted amplicon (AMPL7156123427), the contiguous not-deleted amplicon (AMPL7156321488), and the MLPA probe targeting exon 5 have been reported.

**Figure 3:**
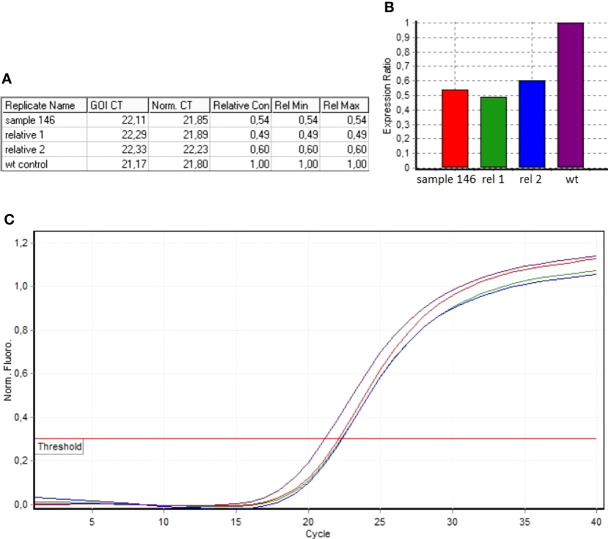
Real-time qPCR results of sample 146 and of MO-affected relatives (Relative 1 and Relative 2). **(A)** The ddCt values relative to sample 146, Relative 1 and Relative 2. **(B)** Ratio values relating to the samples investigated with respect to the wild-type control. **(C)** Amplification curves of both the investigated samples and the wild-type control.

The algorithm was then applied to the 20 samples (Samples 136–155) negative for the presence of *EXT* small mutations (using both the DHPLC/Sanger sequencing and NGS diagnostic approach) and large deletions/duplications (assessed by MLPA). The NGS CNV tool detected a potential heterozygous deletion in Sample 146 mapping outside the MLPA target regions and partially including the intron 4 and the exon 5 of the *EXT2* gene; the deletion has been identified at the amplicon AMPL7156123427, which was characterized by a 'depth (norm.):/ average depth (norm.)' ratio ranging from 42.9% to 45%, depending on the baseline. The novel *EXT2* large deletion—never detected before—is NG_007560.1:g.44146239_44146376 del, NM_207122.1:c.(744-122)-?_804+?del (**Figure 2**). To validate the detection, potential CNV underwent real-time qPCR; as represented in **Figure 3**, the deletion has been confirmed. To further investigate the pathogenicity of the variant, its investigation was extended by real-time qPCR, analyzing two relatives of Sample 146, both affected by MO; the presence of the novel deletion was confirmed (results described in **Figure 3**) in both cases. All other potential CNVs identified were excluded by MLPA or real-time qPCR analyses.

## Discussion

Because NGS is increasingly being used in molecular diagnosis, any gene can be virtually captured and analyzed not only qualitatively but also quantitatively. In the presence of a reliable assay to detect quantitative variants (i.e., CNVs), it is possible to complement or replace traditional methods (i.e., MLPA), overcoming their technical limitations, reducing costs, and improving diagnosis.

Several bioinformatic tools have been developed to detect CNVs from NGS data (3, 4, 17, 18); unfortunately, most are suitable only for whole-genome or whole-exome sequencing data (4), thus not applicable to targeted gene panel that, however, represent the common practice in most diagnostic labs. To facilitate CNVs detection from targeted gene panel, we propose an “easy-to-use” pipeline based on the average amplicon coverage normalization to detect exon, multi-exon, and partial-exon deletions in MO diagnosis, working on NGS data obtained from a two-gene target panel (12.78 kb in size).

The pipeline was validated by analyzing a retrospective cohort of 11 CNV-positive control samples, showing a sensitivity of 97.5% when considering amplicons separately (one amplicon showed a “false negative” result in a deleted region covered by 17 amplicons) and a sensitivity of 100% if we consider that, however, all 11 deletions have been detected. Furthermore, by analyzing a cohort of 20 patients with genetically “undiagnosed” MO, we identified an additional CNV not previously detected by MLPA since located in a region not covered by the probes of the commercial kit; the deleted region includes partial regions of both intron 4 and exon 5 of *EXT2*. The new variant [NM_207122.1: c.(744-122)-?_804+?del] was confirmed by real-time qPCR and also found in two MO-affected relatives.

Overall, the tool was able to detect different deletion sizes occurring at both multi-exonic and exonic level, up to the identification of the novel partial exonic deletion. Unlike the most used CNV tools, our algorithm allowed to identify deletion up to a single NGS amplicon, as is the case of the novel detected variant. As it represents the most challenging aspect (19, 20), the ability to detect small CNVs in target panel-based data makes the CNV algorithm reliable in the diagnostic routine. This is true also considering the high sensitivity, specificity, and accuracy of the method. Regarding this, considering the <75% threshold for a “deletion” call, the 97.5% of sensitivity obtained is in line with the 91%–100% of sensitivity described in previous studies (3, 4, 20). Because the sensitivity does not vary according to the number and type of samples included in the baseline, a 10-sample–randomly selected baseline appears to be enough, thus making the pipeline useful even with few samples available as for the ultra-rare diseases.

In addition, considering specificity (about 96%) and accuracy (about 96%), our results are in line with other CNV detection tools (4, 16, 18, 20). To use the pipeline in the clinical field, limiting the number of false positives makes the diagnosis more efficient as it reduces the need for further validation analyses, thus reducing time and costs. Despite the most of false positives seems to be systematic errors, since observed in the same regions across multiple samples, an attempt to optimize the tool was made by repeating the analyses with a gradually reduced threshold value (initially set to 75%), below which a “deletion” is called, reaching up to 60%. According to our data, the best combination of sensibility, specificity, and accuracy was obtained with a threshold of <75%–70%, below which the sensitivity is not acceptable for clinical use. It should be considered that this evaluation is applicable to the deletion types that we need to detect in the specific clinical context examined (i.e., exon and partial-exon deletions). In the case of larger deletion (in the order of the entire gene), it is recommended to further reduce the CNV detection threshold to minimize the presence of false positives.

Finally, since not limited by the presence of a few MLPA probes covering a partial gene region, CNV detection from NGS data permits a more accurate description of the variants allowing CNV boundaries to be extended to all involved amplicons. The possibility of creating NGS panels to analyze any gene—except for some peculiar gene structure—also makes this tool applicable to all genes for which an MLPA kit is not available, thus increasing the variant detection rate in clinical diagnosis.

As only limitations, a coverage-based tool requires high-quality NGS data especially in terms of “uniformity of coverage”. On the other side, differently by other tools that require uniform NGS data sets (20), less attention can be paid to depth data; this is due to the normalization of the coverage depth (both in query and baseline samples), which does not require similarity of coverage depth across the samples, also making the baseline usable with any investigated sample. As observed in the study, the use of different sample selections does not affect the sensitivity.

In addition to having demonstrated the ability to replace the conventional methods (MLPA), at least in our experience, it must be emphasized as the simplicity of the tool does not require specialized personnel, as well as software purchase, thus making it usable—after a careful evaluation to optimize the parameters—in many diagnostic and research contexts.

## Conclusions

The present study described the use of a simple, free, and easy-to-use method for detecting CNVs from very small NGS panel data, able to identify up to partial-exon deletion. The high sensitivity, specificity, and accuracy provide an alternative diagnostic approach to traditional MLPA, not always usable and with technical limitations, whose versatility allows its use in many research and clinical fields, reducing diagnosis time and costs and improving the variant detection rate.

## Data Availability Statement

The authors acknowledge that the data presented in this study must be deposited and made publicly available in an acceptable repository, prior to publication. Frontiers cannot accept a article that does not adhere to our open data policies.

## Ethics Statement

The studies involving human participants were reviewed and approved by Comitato Etico di Area Vasta Emilia Centro della Regione Emilia-Romagna (CE-AVEC). The patients/participants provided their written informed consent to participate in this study.

## Author Contributions

SC: Conceptualization, methodology, validation, resources, investigation, data curation, formal analysis, writing—original draft, writing—review and editing, supervision, and project administration. EP: Conceptualization, methodology, validation, resources, investigation, data curation, writing—original draft, writing—review and editing, formal analysis, supervision, and project administration. CP: Software, methodology, validation, data curation, and writing—review and editing. MG: Resources and writing—review and editing. ML: Data curation and writing—review and editing. LS: Conceptualization, methodology, writing—review and editing, supervision, and funding acquisition. All authors contributed to the article and approved the submitted version.

## Conflict of Interest

The authors declare that the research was conducted in the absence of any commercial or financial relationships that could be construed as a potential conflict of interest.

## Publisher’s Note

All claims expressed in this article are solely those of the authors and do not necessarily represent those of their affiliated organizations, or those of the publisher, the editors and the reviewers. Any product that may be evaluated in this article, or claim that may be made by its manufacturer, is not guaranteed or endorsed by the publisher.

## Supplementary Material

Supplementary Table 1 List and description of NGS amplicons covering EXT1 and EXT2 (genomic target region, code, target gene, EXT target region).

Supplementary Table 2 Target regions covered by both NGS amplicon and MLPA. For each region, the corresponding MLPA probe and NGS amplicon are indicated.

Supplementary Table 3 NGS metrics for each sample (mean depth, number of mapped reads, uniformity, on-target percentage). In red, the samples excluded due to poor data quality.

Supplementary Table 4 List of samples randomly selected to create each baseline.

Supplementary Table 5 Results of the CNV detection on the 11 positive controls. For each baseline sample size (10, 30, 50, 100), results using all the 5 iterations are reported. All depth(normalized)/average depth (normalized) ratio <75% are reported. Ratio >75% are reported only in case of discrepancy between MLPA and NGS CNV pipeline. The delete regions are highlighted in green. For the amplicons that overlap with an MLPA target region, the whole column is highlighted in grey.

Supplementary Table 6 NGS CNV pipeline effectiveness in relation to the different baselines: true positive (TP), true negative (TN), false positive (FP), false negative (FN), sensitivity, specificity and accuracy are reported for each baseline.

Supplementary Table 7 Sensitivity, specificity, and accuracy calculated according to different baseline sample sizes and different CNV-detection thresholds.

Supplementary Figure 1 CNV distribution in EXT1 and EXT2 genes (data from LOVD - MOdb).EXT1 large deletions; exon 1 is specified separately as, being quite long, it is comparable to a multi-exon deletion.EXT2 large deletions
