# Supplementary figures and images for: An Easy-to-Use Approach to Detect CNV From Targeted NGS Data: Identification of a Novel Pathogenic Variant in MO Disease
Source: Front Endocrinol (Lausanne). 2022 Jun 28;13:874126. doi: 10.3389/fendo.2022.874126 (PMC9273874; doi:10.3389/fendo.2022.874126)

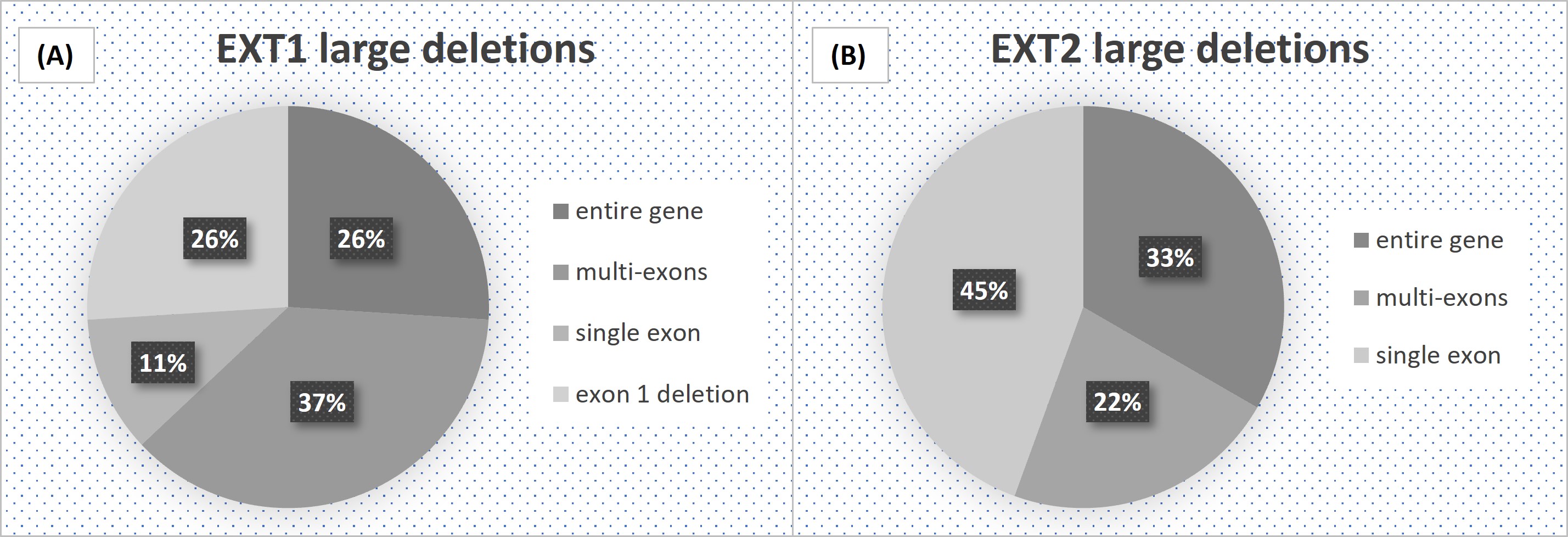

Supplement: Supplementary Figure 1 — CNV distribution in EXT1 and EXT2 genes (data from LOVD - MOdb).EXT1 large deletions; exon 1 is specified separately as, being quite long, it is comparable to a multi-exon deletion.EXT2 large deletions [file Image_1.jpeg]
